# Co-Expression Network Analysis Suggests PacC Transcriptional Factor Involved in *Botryosphaeria dothidea* Pathogenicity in Chinese Hickory

**DOI:** 10.3390/jof11080580

**Published:** 2025-08-04

**Authors:** Dong Liang, Yiru Jiang, Wei Ai, Yu Zhang, Chengxing Mao, Tianlin Ma, Chuanqing Zhang

**Affiliations:** College of Advanced Agricultural Sciences, Zhejiang Agriculture and Forest University, Hangzhou 311300, China; liangdong0309@163.com (D.L.);

**Keywords:** *Botryosphaeria dothidea*, Chinese hickory tree, co-expression network, PacC transcriptional factor

## Abstract

*Botryosphaeria dothidea* is the causative agent of Chinese hickory trunk canker, which poses significant threat to the production of Chinese hickory (*Carya cathayensis* Sarg.). Previous studies reported that endophytic–pathogenic phase transition, also referred to as latent infection, plays an important role in the interaction of *Botryosphaeria dothidea* with various host plants, including Chinese hickory. However, the mechanism underlying this phase transition is not well understood. Here, we employed RNA-Seq to investigate transcriptional changes in *B. dothidea* during its phase transition upon interaction with Chinese hickory. A co-expression network was generated based on 6391 differentially expressed genes (DEGs) identified from different infection stages and temperature treatments. One co-expressed module was found that highly correlated with temperature treatments which simulated conditions of *B. dothidea* latent infection in the field. Subsequently, 53 hub genes were detected, and gene ontology (GO) enrichment analysis revealed three categories of enriched GO terms: transmembrane transport or activity, ion homeostasis or transport, and carbohydrate metabolism. One PacC transcriptional factor (BDLA_00001555, an ambient pH regulator), and one endo-β-1,3-glucanase (BDLA_00010249) were specifically upregulated under temperature treatments that corresponded with the activation stage of *B. dothidea*’s pathogenic state. The knockout mutant strain of BDLA_00001555 demonstrated defective capability upon the activation of the pathogenic state. This confirmed that BDLA_00001555, the PacC transcriptional factor, plays an important role in the latent infection phase of *B. dothidea*. Our findings provide insights into the pathogenic mechanism of Chinese hickory trunk canker disease.

## 1. Introduction

**Figure 1:**
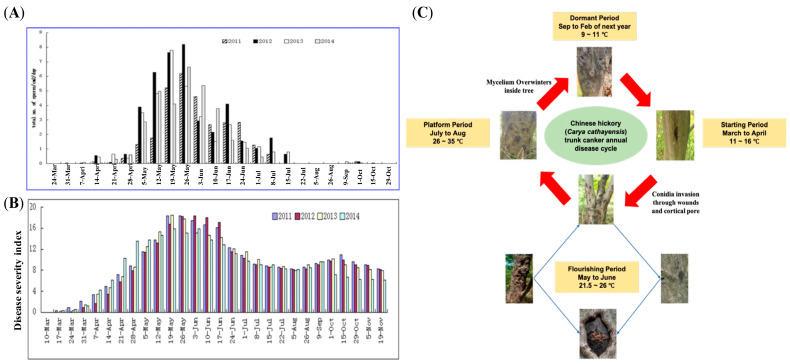
Comprehensive overview of Chinese hickory trunk canker incidence. (**A**) Investigation of spore concentration for years 2014 to 2018. (**B**) Investigation of disease severity index for years 2014 to 2018. (**C**) Disease cycle of Chinese hickory trunk canker.

Chinese hickory (*Carya cathayensis* Sarg.) is a member of the Juglandaceae family. It is an economically important nut tree in China. In the past decade, approximately 90% of hickory orchards in China have been significantly affected by Chinese hickory trunk canker. This is a yield-devastating disease caused by the ascomycete fungus *Botryosphaeria dothidea* [1], a major pathogen responsible for cankers or dieback in a broad range of woody hosts [2]. *B. dothidea* penetrates trunk–bark tissue, initially forming small elliptical lesions. These lesions enlarge and develop into sunken and elongated cankers, ultimately resulting in typical canker symptoms [3,4,5,6]. In a previous work [7], we installed spore traps and meteorological parameter recorders in fields (Lin’an district, China; 29 to 31° N and 118 to 120° E) to monitor dynamic changes in the airborne spore flux of *Botryosphaeria dothidea* and the timing of Chinese hickory canker disease occurrence. Investigations spanning eight years (from 2011 to 2018) revealed that canker symptoms appear approximately one week before the release of spores in fields (Figure 1A). Based on this research, it has been postulated that the disease cycle of Chinese hickory trunk canker can roughly be divided into the following four distinct periods [8] (Figure 1B): (i) the dormant period, when *B. dothidea* serves as an endophyte following its initial penetration into the tree trunk and overwinters within the trunk as mycelium (typically from September to February, when the average temperature is 10 °C); (ii) the starting period, when *B. dothidea*’s mycelium breaks through the bark in early spring (from March to April, when the average temperature is 15 °C), forming ulcerous lesions as well as pycnidia on the lesions; (iii) the flourishing period, when pycnidiospores are released from the pycnidia and are spread by the wind between hosts, causing subsequent infections and resulting in the substantial emergence of lesions and disease outbreaks (the peak spore dispersal in orchards occurs from May to June, when the average temperature is 25 °C); and (iv) the platform period, when the disease progression gradually reduces and when no further lesions appear because of comparatively high temperatures (typically from July to August, when the average temperature is 35 °C). From September onwards, *B. dothidea* reverts to an endophytic state, surviving the winter months as mycelium within the tree, and awaiting the return of conducive conditions in the subsequent spring. This highlights a remarkable shift in *B. dothidea*’s life cycle, toggling between an endophytic existence and pathogenicity during its engagement with Chinese hickory trees. This phenomenon is supported by the research of [2], which highlighted that a detailed mechanism of *B. dothidea*’s state of transition, or latent infection, is still unclear. Consequently, we proposed the following hypothesis: a moderate temperature increase (from 15 °C to 25 °C) is a required environmental factor for *B. dothidea* to transition from being endophytic to being pathogenic.

Transcriptome sequencing is an efficient approach when identifying pathogenicity-associated genes at a genomic level. The transcriptomes of grapes infected with *B. dothidea*, *Lasiodiplodia theobromae*, and *Neofusicoccum parvum* have been sequenced in previous research to identify multiple virulence-related genes specific to *B. dothidea* [9]. The WGCNA method has been widely employed to identify the key genes associated with traits of interest, including research based on a large-scale sample set [10]. Ref. [11] screened out five candidate resistance genes to wheat stripe rust using the WGCNA approach. A WGCNA analysis was used to detect the transcriptional factors that regulate pathogen-related genes. One C_2_H_2_ transcriptional factor, PsCZF3, was observed to control the transcription of 35 RxLR effectors in *Phytophthora sojae* [12]. A WGCNA analysis is a powerful tool for the identification of genes associated with *B. dothidea*’s latent infection phase.

The PacC transcriptional factor is a dominant component of an ambient pH-responsive regulatory system. It is widely distributed in multiple filamentous fungi, including *Aspergillus nidulans*, *Saccharomyces cerevisiae*, *Candida albicans*, and *Magnaporthe oryzae* [13]. The ambient pH signaling pathway, namely the Pal/Rim-pH pathway, is composed of PacC and six Pal proteins (PalA, PalB, PalC, PalF, PalH, and PalI). pH signals originating from the extracellular environment govern the expression of PacC and mediate the transitions between its three distinct protein isoforms [14,15,16,17], activating/inactivating the downstream alkaline- or acid-expressed genes through the binding of the core consensus sequence 5′-GCCARG-3′ [18]. PacC has also been demonstrated to affect the pathogenicity of various phytopathogenic fungi. A *PacC* knockout of *Aspergillus* and *Penicillium* species contributed to a decreased virulence [19,20], but a *PacC* knockout of *Magnaporthe oryzae* failed to switch between biotrophic and necrotrophic growth, and the disruption of *PacC* enhanced the pathogenicity of *Fusarium oxysporum* [21,22]. PacC regulates the expression of multiple downstream target genes. For example, [23] demonstrated that the Δ*pacC* mutant of *Aspergillus fumigatus* exhibited significantly reduced activity in epithelial cell detachment assays and completely lacked protease activity in gelatin degradation assays, indicating that PacC promotes host invasion by regulating expression of protease genes [22]. In *Botrytis cinerea*, PacC controls the expression of numerous secondary metabolism-related enzymes, including key polyketide synthase genes (*Bcpks4*, *Bcpks5*, and *Bcpks11*) involved in sporulation and pathogenicity. Deletion of PacC resulted in significant alterations of secondary metabolic pathways, suggesting that PacC modulates virulence by regulating specific metabolite biosynthesis. Moreover, in *Trichoderma virens*, PacC regulates multiple ion transporter genes that are upregulated under alkaline pH conditions, such as the P-type ATPase Ena1 and the ZIP zinc transporter, facilitating adaptation to alkaline environments [24]. The co-expression of an iron siderophore transporter with a non-ribosomal peptide synthetase gene under alkaline conditions implies the potential role of PacC in iron metabolism regulation. Conversely, the high-affinity glucose transporter is also upregulated at an alkaline pH but is not regulated by PacC, indicating specificity in PacC-mediated transcriptional control [25]. This indicates that PacC serves multiple roles in the pathogenicity of phytopathogenic fungi, but the function of PacC in the temperature-regulated latent infection phase of *B. dothidea* remains unknown.

In this study, we aimed to explore the underlying mechanisms of *B. dothidea*’s latent infection phase by constructing a co-expression network associated with temperature treatments that simulated environmental conditions in the field. We observed a number of major facilitator transporters, sugar transporters, enzymes related to carbohydrate metabolism, and transporters responsible for multiple ions such as iron and potassium. We also observed a notable upregulation of one PacC transcription factor during *B. dothidea*’s transition from an endophytic to a pathogenic state. A mutation of this PacC gene disrupted the progression of the latent infection phase and significantly attenuated the fungus’s pathogenicity, underscoring its crucial role in the infection process. The results of this study provide important information regarding the molecular mechanism of *B. dothidea*’s latent infection phase, helping to elucidate the endophytic–pathogenic transition of *B. dothidea*.

## 2. Results

### 2.1. In Vitro Simulation of B. dothidea’s Latent Infection Phase Using Chinese Hickory

**Figure 2:**
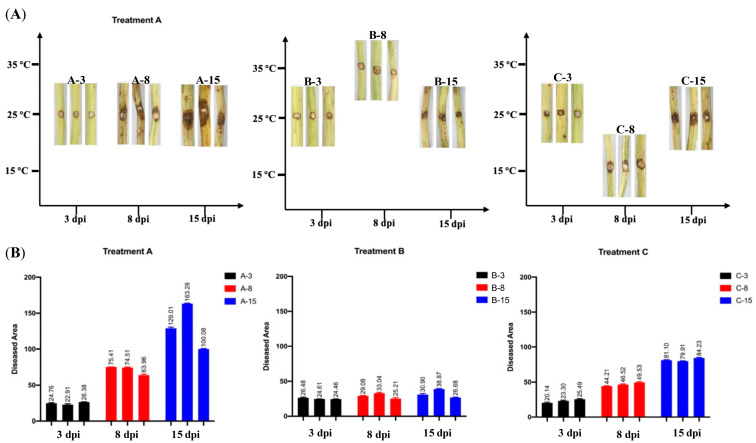
Disease reactions of wild-type Chinese hickory trunk incubated with BDLA16-7, the wild-type strain of *B. dothidea*. (**A**) Photographs showing the disease reactions of Chinese hickory trunk under indicated temperature treatments: A-3, A-8, and A-15 represent 3, 8, and 15 dpi of Treatment A; B-3, B-8, and B-15 represent 3, 8, and 15 dpi of Treatment B; and C-3, C-8, and C-15 represent 3, 8, and 15 dpi of Treatment C. (**B**) The disease symptoms on trunks of Chinese hickory to *B. dothidea* strain BDLA16-7. ImageJ (version 1.53) was used to calculate the diseased area. Different letters above bars indicate significant differences (*p* < 0.01 using one-way analysis of variance [ANOVA]).

The molecular mechanism underlying the latent infection phase of *B. dothidea* was elucidated by cultivating inoculated Chinese hickory seedlings in growth chambers. Precisely controlled temperatures were used to simulate the environmental conditions associated with the primary infection stages of Chinese hickory trunk canker disease in the field. As described in the Methods and Materials section and Figure 2A, all inoculated seedlings were initially cultivated at 25 °C for 3 days (corresponding with samples A-3, B-3, and C-3), which is the optimal growth temperature for *B. dothidea* to ensure its successful colonization [26]. The inoculated seedlings were then divided into the following three groups for separate cultivation: (i) Treatment A (samples A-8), where the samples in this group were cultivated at a constant temperature of 25 °C for 5 days to serve as the control group; (ii) Treatment B (samples B-8), where the samples in this group were cultivated at 35 °C for 5 days to serve as a control to observe the changes in *B. dothidea*’s pathogenicity upon its shift back to normal temperatures after exposure to high-temperature stress; and (iii) Treatment C (samples C-8), where the samples in this group were cultivated at 15 °C for 5 days to serve as the treatment group corresponding with the environmental temperature fluctuations implicated in the activation of *B. dothidea*’s transition from an endophytic to a pathogenic state. Subsequently, all inoculated seedlings were transferred to a 25 °C environment for an additional 7 days of cultivation (corresponding with samples A-15, B-15, and C-15).

As Figure 2 reveals, no significant differences in *B. dothidea*’s pathogenicity were detected in samples A-3, B-3, and C-3 (corresponding with Treatments A, B, and C, respectively) for the first 3 days post-inoculation (dpi). The diseased areas ranged from 20.14 to 26.48. This result was expected because all samples underwent the same thermal treatment during this stage. The A-8 sample exhibited diseased regions averaging approximately 63.96 to 75.41 at 8 dpi, markedly exceeding those observed in both the Treatment B (B-8; 25.21 to 33.04) and Treatment C (C-8; 44.21 to 49.53) samples. This suggested that an inappropriate temperature negatively affected *B. dothidea*’s pathogenicity. The A-15 sample displayed the most extensive disease severity at 15 dpi, with an average diseased area of 100.08 to 163.28. This was notably higher than that of Treatment B (B-15; 26.68 to 38.87). Slightly larger diseased areas were observed in the A-15 sample compared with those of Treatment C (C-15; 79.91 to 81.10). This implies that *B. dothidea*’s pathogenicity could be reactivated upon a return to 25 °C, even after being subjected to a temperature treatment of 15 °C. This suggests the occurrence of *B. dothidea*’s latent infection phase during its invasion of hickory trees.

### 2.2. RNA-Seq Data Processing and Identification of DEGs

The samples described above were collected for RNA sequencing, each with three biological replicates. Table 1 presents a range of 33,063,322 to 41,068,181 pairs of raw reads generated across all samples. Clean reads of 83% to 92% were obtained after filtering out low-quality reads. A range of 372,589 to 2,488,240 pairs of reads was mapped to the genome of *B. dothidea* BDLA16-7, as published in our previous research [27].

A principal component analysis (PCA) was employed to evaluate the overall differences among the samples of this study. As depicted in Figure S1, samples A-3, B-3, and C-3 were closely clustered together, likely because of exposure to the identical condition of 25 °C for 3 days. Samples A-8, A-15, and C-15 exhibited minimal separation, suggesting that the infection process of *B. dothidea* could recover to normalcy after a temperature treatment of 15 °C. Samples B-8, B-15, and C-8 revealed clear differentiation, both from other samples and between themselves, suggesting that the pathogen’s infection process encountered difficulty in returning to normalcy after a temperature treatment at 35 °C. Samples C-3, C-8, and C-15 were subsequently chosen to explore the latent infection mechanisms of *B. dothidea*.

**Figure 3:**
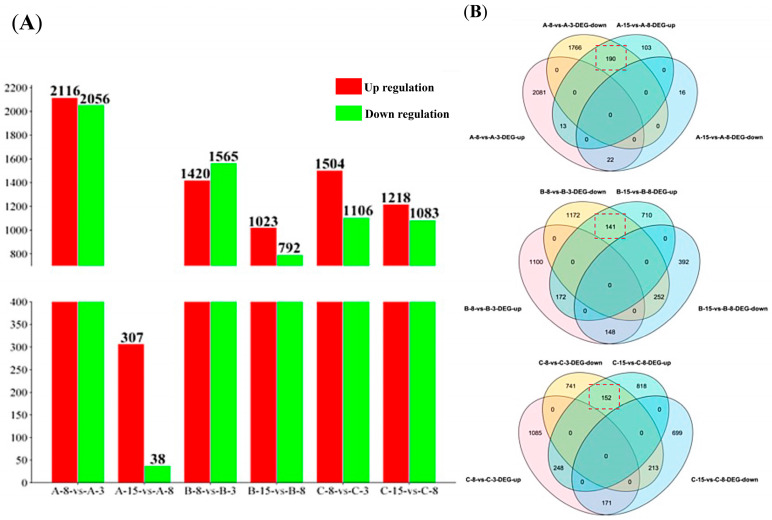
Overview of differentially expressed genes (DEGs) under Treatments A, B, and C. (**A**) Quantification of DEGs at 3, 8, and 15 days post-incubation (dpi) under conditions of Treatments A, B, and C; (**B**) Venn diagram showing commonly and specifically upregulated and downregulated DEGs under conditions of Treatments A, B, and C. Red dashed box marked DEGs that downregulated at 8 dpi but upregulated at 15 dpi.

The identification of differentially expressed genes (DEGs) is based upon a comparison of samples from later time points with those from earlier time points (such as 8 dpi compared with 3 dpi, or 15 dpi compared with 8 dpi). As presented in Figure 3A and Table S1, 4172 DEGs (2116 upregulated and 2056 downregulated) were identified in the A-8 to A-3 comparison, 345 DEGs (307 upregulated and 38 downregulated) were identified in the A-15 to A-8 comparison, 2985 DEGs (1420 upregulated and 1565 downregulated) were identified in the B-8 to B-3 comparison, 1815 DEGs (1023 upregulated and 792 downregulated) were identified in the B-15 to B-8 comparison, 2610 DEGs (1504 upregulated and 1106 downregulated) were identified in the C-8 to C-3 comparison, and 2301 DEGs (1218 upregulated and 1083 downregulated) were identified in the C-15 to C-8 comparison. After the removal of redundancy from these DEGs, we obtained a DEG gene set comprising 6391 DEGs (Table S2).

Through a simple comparison of the DEGs (Figure 3B), we observed 190 DEGs downregulated in A-8 but upregulated in A-15, 22 DEGs upregulated in A-8 but downregulated in A-15, 141 DEGs downregulated in B-8 but upregulated in B-15, 145 DEGs upregulated in B-8 but downregulated in B-15, 152 DEGs downregulated in C-8 but upregulated in C-15, and 171 DEGs upregulated in C-8 but downregulated in C-15.

### 2.3. A Co-Expression Network Construction Revealing the Genes Associated with B. dothidea’s Latent Infection Phase in Chinese Hickory

A weighted gene co-expression network analysis (WGCNA) was used to construct a co-expression network to identify the genes specifically associated with *B. dothidea*’s latent infection phase in Chinese hickory. This was based on the 6391 previously identified DEGs. The WGCNA soft-threshold β was optimized at 9, coinciding with the scale-free topology fit index’s first attainment of 0.9. This was complemented by a minimum module size threshold of 50 (Figure S2).

**Figure 4:**
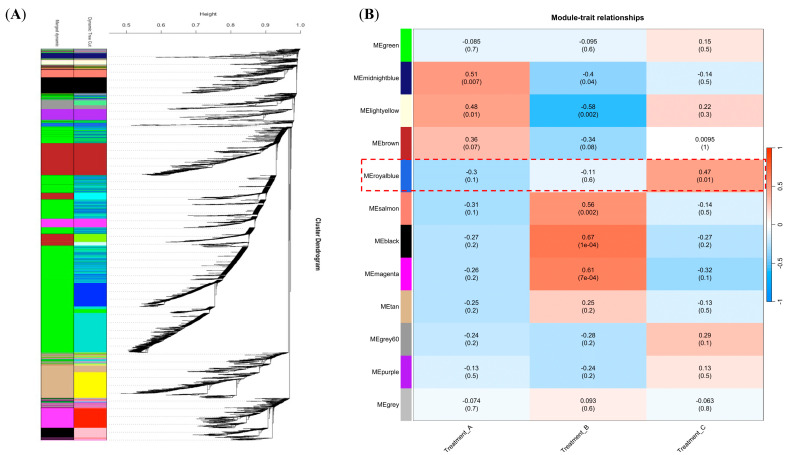
Construction of gene co-expression network and module detection by gene cluster dendrograms. (**A**) Module detection based on gene co-expression network. (**B**) Module–trait associations estimated using correlations between module eigengene and conditions of Treatments A, B, and C.

**Figure 5:**
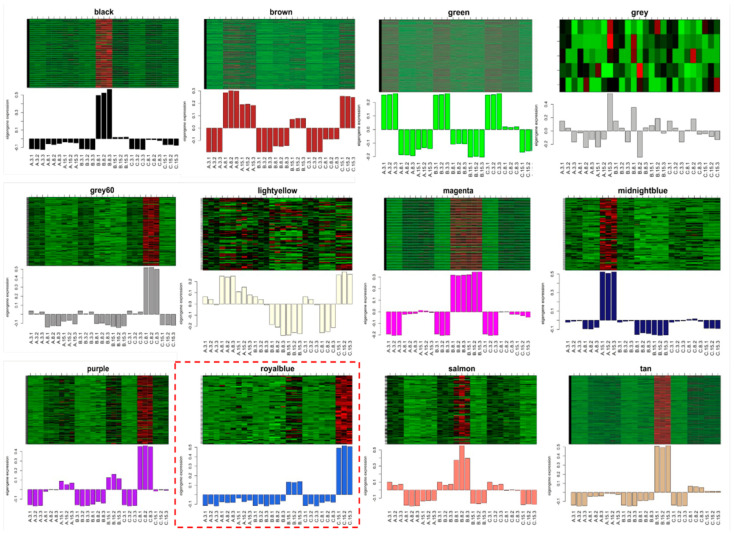
Expression patterns of eigengenes for each module determined by the gene co-expression network.

All DEGs were grouped into 12 co-expressed modules from a correlation analysis based on their expression levels (Figure 4A and Table S3). DEGs with stronger correlations were grouped together within the same module. Different colors were used to differentiate modules; grey modules were used to indicate the genes that could not be assigned to any specific module. The green (2986), brown (918), and tan modules harbored the highest number of DEGs, whereas the royal-blue (70), light-yellow (76), and midnight-blue (93) modules exhibited the lowest number of DEGs. We estimated module-trait associations by correlating the expression profiles of module eigengenes with the temperature conditions used for Treatments A, B, and C to explore the link between the co-expression modules and *B. dothidea*’s latent infection phase. Figure 4B reveals that the midnight-blue (R_A_ = 0.51; *p* = 0.007) and light-yellow (R_A_ = 0.48; *p* = 0.01) modules exhibited a significant association with the temperature setting of Treatment A. Conversely, the black (R_B_ = 0.67; *p* = 0.0001), magenta (R_B_ = 0.61; *p* = 0.0007), and salmon (R_B_ = 0.56; *p* = 0.002) modules were closely linked to the temperature setting of Treatment B. As the temperature settings of Treatment C closely mimicked the actual environmental conditions conducive to *B. dothidea*’s latent infection phase in the field, we focused on the royal-blue module. This was significantly correlated with Treatment C (R_C_ = 0.47; *p* = 0.01). The expression pattern of the eigengenes within the royal-blue module exhibited significant upregulation—specifically, at 15 dpi—under Treatment C (Figure 5). This suggests that the genes assigned to the royal-blue module might play a crucial role in *B. dothidea*’s transition from an endophytic to a pathogenic state. The royal-blue module was selected as the target module for the subsequent analysis.

### 2.4. The Identification of the Hub Genes in the Royal-Blue Module and the Enrichment Analysis

The gene significance (GS) value quantifies the association between individual genes and interesting traits. The module membership (MM) value represents the correlation between module eigengenes and gene expression profiles [10]. We calculated the GS and MM values for each gene in the royal-blue module to investigate the potential key genes—also called the hub genes—to determine *B. dothidea*’s latent infection phase. In this study, we followed the threshold of GS and MM proposed by previous studies [28,29]. A total of 52 key genes were identified in the royal-blue module by setting the thresholds GS > 0.2 and MM > 0.8. The expression profiles of these key genes were similar to the eigengenes of the royal-blue module (Table S4).

**Figure 6:**
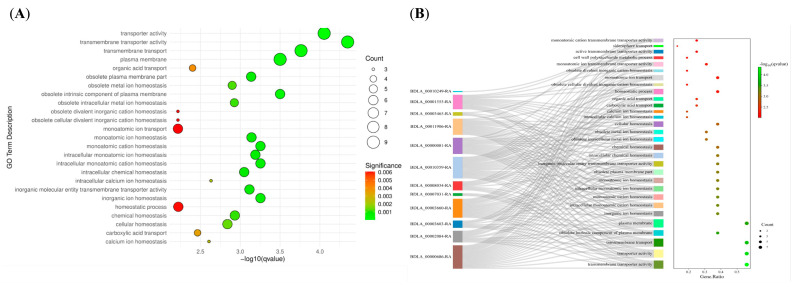
Overview of GO enrichment analysis of genes assigned to royal-blue module. (**A**) Enriched GO terms. (**B**) Chord diagram showing correspondence relationship between genes and corresponding enriched GO terms.

GO annotation and enrichment analyses were conducted to obtain deeper insights into the functions of these hub genes. As presented in Table S5 and Figure 6A, the enriched GO terms were roughly categorized into the following three groups: (i) transmembrane transport or activity (e.g., ‘transporter activity’, ‘transmembrane transporter activity’, and ‘transmembrane transport’); (ii) metal ion homeostasis or transport (e.g., ‘metal ion homeostasis’, ‘monoatomic ion homeostasis’, and ‘calcium ion homeostasis’); and (iii) carbohydrate metabolism (e.g., ‘cell wall polysaccharide metabolic process’ and ‘carboxylic acid transport’). In total, 13 hub genes were discovered to be assigned to enriched GO terms (Figure 6B). BDLA_00011906 and BDLA_00000081 possessed the MFS_1 (PF07690) domain; both have been annotated as siderophore iron transporters and sugar transporters using Nr annotation. We identified BDLA_00001555, which harbors the C_2_H_2_-type zinc finger domain (PF00096). This was assigned to diverse enriched GO terms; it has previously been annotated as a PacC transcriptional factor primarily associated with pH regulation. Two hydrolases (BDLA_00010359 and BDLA_00000686) and carbohydrate metabolism-related genes (BDLA_00010249 and BDLA_00003465), enriched in the GO terms, were associated with carbohydrate metabolism.

**Figure 7:**
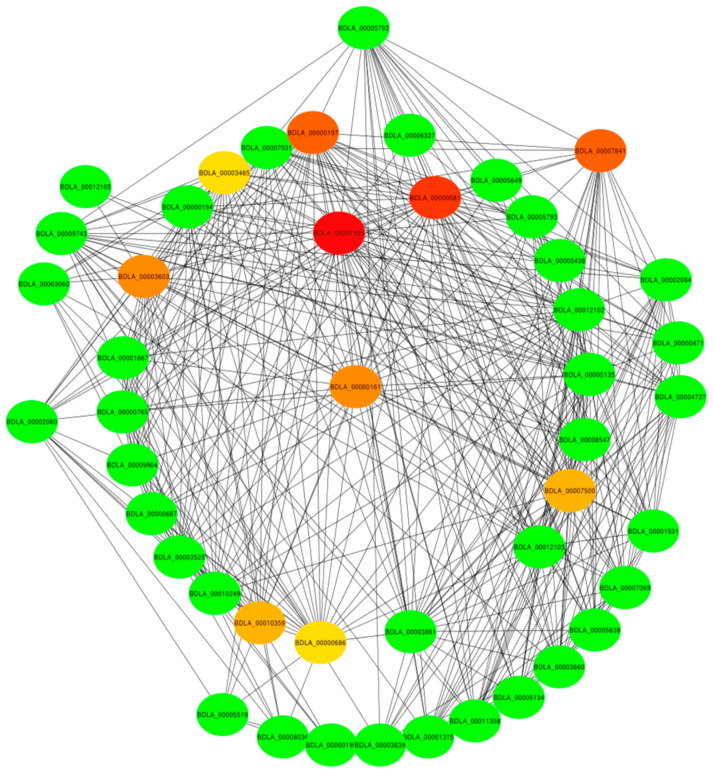
Co-expression network analysis for hub genes of royal-blue module.

In a co-expression network, hub genes are characterized by having a considerably greater number of connections or interactions compared with non-hub genes within the network. This indicates that hub genes are often pivotal for the governance of multiple biological processes and are crucial for the overall functionality and robustness of the network [30]. In this study, we assessed the connectivity of hub genes in a co-expression network using the Cytoscape Hubba plugin. As presented in Figure 7, the top 10 genes with the highest connectivity were BDLA_00001555 (PacC transcription factor), with 22 connections; BDLA_00000081 (major facilitator transporter), with 16 connections; BDLA_00000197 (2OG-Fe(II) oxygenase); BDLA_00007841 (carbon–nitrogen hydrolase); BDLA_00000161 (ZIP zinc transporter); BDLA_00003603 (MIP aquaporin); BDLA_00007500 (major facilitator transporter); BDLA_00010359 (cation transport ATPase); BDLA_00000686 (Mg^2+^-dependent Ca^2+^/ATP hydrolase); and BDLA_00003465 (1,3-beta-glucan elongating enzyme). We posited that these hub genes might be involved in the activation of *B. dothidea*’s pathogenic state.

### 2.5. Validation of Hub Genes Using RT-PCR

**Figure 8:**
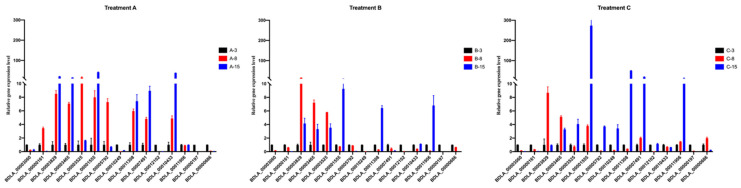
Relative expression levels of 15 hub genes, with the highest expression level in RNA-seq data in the royal-blue module. The different letters denote significant differences with *p* < 0.05. A-3, A-8, and A-15 represent 3, 8, and 15 dpi of Treatment A; B-3, B-8, and B-15 represent 3, 8, and 15 dpi of Treatment B; and C-3, C-8, and C-15 represent 3, 8, and 15 dpi of Treatment C.

Of the 52 hub genes, 15 were selected for the RT-PCR validation based on their highest expression levels in the RNA-Seq sample from C-15 (15 dpi; Treatment C). As presented in Figure 8 and Table S4, seven genes were significantly upregulated. The expression levels ranged from 3- to 270-fold in sample C-15, which comprised BDLA_00001555 (PacC transcription factor), BDLA_00005792 (containing a cysteine and histidine-rich domain), BDLA_00010249 (featuring a Glyco_hydro_81 domain), BDLA_00003525 (containing a Sugar_tr domain), BDLA_00011308 (with a Glyco_hydro_64 domain), BDLA_00011308, and BDLA_00007491 (with an MFS_1 domain). When comparing the expression pattern of these genes in Treatments A and B, we observed that BDLA_00003525, BDLA_00005792, BDLA_00010249, and BDLA_00010249 were specifically upregulated in sample C-15. Although BDLA_00001555 exhibited a significant upregulation (approximately 10-fold) in Treatments A and B, its expression was markedly increased (up to 270-fold) in Treatment C. We ascertained that these five candidate genes were likely to be closely involved or regulated in *B. dothidea*’s latent infection phase.

### 2.6. PacC Transcriptional Factor Is Important for B. dothidea’s Latent Infection Phase

**Figure 9:**
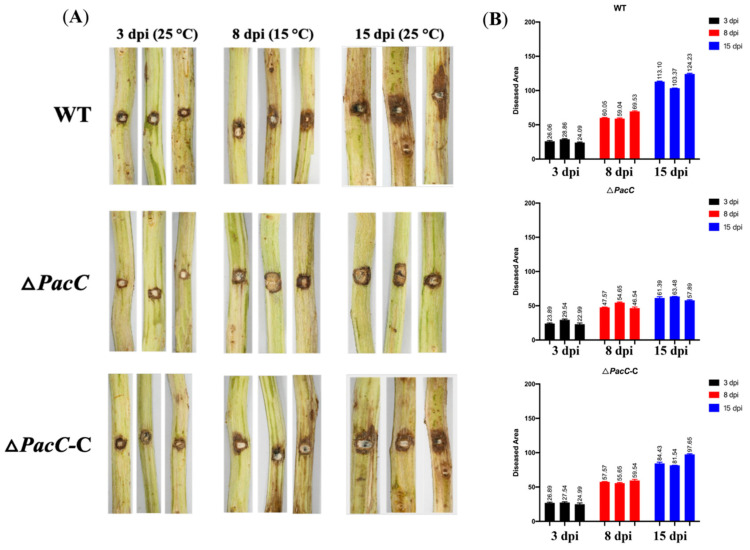
Disease responses of Chinese hickory trunk to *PacC* knockout mutant and wild-type *B. dothidea* strain BDLA16-7 under Treatments A, B, and C. (**A**) Disease symptoms of inoculated trunks. (**B**) Diseased areas caused by *PacC* knockout mutant and wild-type *B. dothidea* strain BDLA16-7 were calculated and statistically analyzed by ImageJ.

We observed from the RT-PCR results that BDLA_00001555—the PacC transcriptional factor—was dramatically upregulated at 15 dpi for Treatment C. We evaluated the pathogenicity of the PacC mutant strain (Δ*PacC*) compared with the wild-type strain (BDLA16-7) to validate its role in *B. dothidea*’s latent infection phase. We complemented the strain (Δ*PacC*-C) using infection assays following the temperature conditions of Treatment C. No significant differences were observed in the Δ*PacC* (22.99 to 29.54), wild-type (24.09 to 28.86), and complemented (24.99 to 27.54) strains at 3 dpi (Figure 9). The diseased areas caused by the wild-type (59.04 to 69.53) and complemented (55.65 to 59.54) strains were significantly larger than that of Δ*PacC* (46.54 to 54.65) at 8 dpi. A similar phenomenon was observed at 15 dpi. We posited that the absence of the PacC transcriptional factor led to the impaired ability of *B. dothidea* to transition from an endophytic to a pathogenic state. Meanwhile, compared to Δ*PacC*, the expression level of *PacC* in the Δ*PacC*-C strain was significantly restored, approaching that of the wild-type strain (Figure S3).

We measured the growth rates of the Δ*PacC*, wild-type, and complemented strains under different temperature treatments of 15 °C, 25 °C, and 35 °C to explore if the diminished pathogenicity of the Δ*PacC* strain on Chinese hickory trunks under Treatment C could be attributed to its growth impairment. The growth rate of Δ*PacC* was only slightly slower than that of the wild-type and complemented strains. This suggested that the reduced pathogenicity of Δ*PacC* was not primarily caused by a growth defect.

## 3. Discussion

Chinese hickory is easily susceptible to damage from various fungal pathogens, most notably *Botryosphaeria dothidea*. This pathogen is responsible for hickory trunk canker, a disease that inflicts severe damage and leads to significant losses in Chinese hickory production [31]. Approximately 90% of the orchard trees in the Zhejiang and Anhui provinces of China have been affected by this disease [7,32]. Investigating the molecular mechanisms underlying the *B. dothidea*–Chinese hickory interaction is necessary for the implementation of environmentally friendly disease control measures. *B. dothidea* has a global distribution and infects numerous managed and natural woody plants. It is responsible for inducing a range of disease manifestations, including cankers on twigs, branches, and stems as well as ring rot, tip necrosis, and dieback of branches [6,33] *B. dothidea* has also been implicated in the development of ring rot and canker diseases in both apple and pear trees [34,35,36], and the occurrence of branch cankers induced by *B. dothidea* in citrus plants has been documented. This pathogen has also been reported to cause shoot blight in pistachio trees [33]. Previous research has delved into *B. dothidea*’s pathogenic mechanisms. The authors of [37] discovered that the deletion of an autophagy-related gene in *B. dothidea*, *BdATG8*, significantly impaired *B. dothidea*’s pathogenicity on apple fruit. Knockout mutants of phosphatase complex Nem1/Spo7 failed to induce any characteristic disease symptoms on both apple fruit and twigs [38]. *B. dothidea* is distinguished by its biphasic lifestyle; it commonly inhabits host plants as an endophyte within asymptomatic tissues for prolonged periods and switches to a pathogenic state under special environmental conditions [2,6]. Our previous long-term field investigation [7] identified that *B. dothidea* exists in the form of an endophyte after invading Chinese hickory trunks until the following February. Subsequently, *B. dothidea* undergoes a phenotypic transformation into overt pathogenicity as temperatures gradually rise from 15 °C to 25 °C (around May to June), provoking the onset of canker formation alongside extensive spore dissemination. This metamorphosis from endophyte to pathogen underscores the pivotal role of temperature in orchestrating the disease cycle of *B. dothidea* [2]. Understanding how key genes or factors drive *B. dothidea*’s transition from endophytic to pathogenic states is crucial when determining *B. dothidea*’s infection processes, but this understanding remains unclear.

### 3.1. Hub Genes Predicted by Co-Expression Analysis Were Associated with B. dothidea’s Infection and Metal Ion Homeostasis or Transport

In this study, we used three groups of infection assays (Treatments A, B, and C) to simulate *B. dothidea*’s infection of Chinese hickory under field conditions. Interactive samples of Treatments A, B, and C were collected for RNA-Seq. A co-expression network was established based on the 6391 identified DEGs using the WGCNA R package (version 1.73) to precisely target the genes associated with *B. dothidea*’s latent infection phase. The co-expression network consisted of 12 modules. One module, the royal-blue module, was closely correlated with the temperature conditions required for *B. dothidea*’s latent infection phase (Treatment C) (*R*_treatment_c_ = 0.47; *p* = 0.01). This module included 70 genes; 53 were characterized as hub genes, which usually serve an important role in the biological regulatory network. The GO enrichment analysis identified enriched GO terms, which were primarily categorized into the following three groups: transmembrane transport or activity, ion homeostasis or transport, and carbohydrate metabolism. Of the 53 hub genes, 12 were assigned to these enriched terms (Table S5). We observed that two major facilitator superfamily proteins (BDLA_00000081 and BDLA_00011906), one sulfate permease (BDLA_00003660), one hydrolase (BDLA_00000686), and one PacC transcriptional factor (BDLA_00001555) were assigned to the enriched GO terms related to metal ion homeostasis or transport. Hosts employ strategies to limit or selectively concentrate metals, posing challenges to microbial proliferation; this is necessary in pathogenic fungi for metal homeostasis control and acquisition [39]. The maintenance of a metal ion equilibrium is crucial for the growth and virulence of plant pathogens. The authors of [40] reported that the deletion of *hapX*, a key iron regulator of iron homeostasis, impaired the growth of *Fusarium oxysporum* under iron-depleted conditions and led to a decreased pathogenicity of the mutated strains on tomato plants. We posit that the hub genes enriched in the GO terms of metal ion homeostasis or transport appear to play an important role in the activation of *B. dothidea*’s pathogenic state.

### 3.2. PacC Transcription Factor

Combined with the results of the RT-PCR and network connectivity analyses, we observed that BDLA_00001555—the PacC transcription factor—was specifically upregulated in sample C-15. This corresponded with the activation stage of *B. dothidea*’s pathogenic state. Infection assays revealed that the pathogenicity of Δ*PacC* was impaired after 5 days of temperature treatment at 15 °C, supporting the hypothesis that this PacC transcription factor plays an important role in *B. dothidea*’s latent infection phase. PacC transcription factors typically sense and respond to fluctuations in the ambient pH, influencing diverse biological processes in pathogenic fungi and affecting their pathogenicity. The authors of [12,41] observed that cooling a whole leaf instigated rapid acidification within a potato’s cytosolic environment. The authors of [42] discovered that a high temperature induced an electrical response that caused a decrease in cytoplasmic pH and an increase in apoplastic pH in *Zea mays*. Phytochrome-interacting factor 4 (PIF4) in *Arabidopsis* is known to stimulate the expression of auxin biosynthesis genes, consequently causing elevated acidity levels in aerial tissues in response to higher temperatures [43]. An alteration in the extracellular pH serves as a signaling mechanism for the regulation of diverse physiological processes in plants, including metal ion transport [44]; this was also observed in the enriched GO terms identified in this study. We speculated that temperature changes during the latent infection period of *B. dothidea* could impact pH levels within Chinese hickory. However, the downstream targets of the *B. dothidea* PacC transcription factor remain to be elucidated through further in-depth investigations.

### 3.3. Carbohydrate Metabolism Enzymes

One endo-β-1,3-glucanase (BDLA_00010249) containing the Glyco_hydro_81 domain and one enzyme (BDLA_00003465) with an X8 domain, involved in trans-glycosylation during 1,3-beta-glucan elongation in the cell wall, were identified to be enriched in GO terms such as the ‘carbohydrate metabolic process’ and the ‘carbohydrate biosynthetic process’. BDLA_00010249 was observed to be specifically upregulated 15 days post-inoculation (dpi) under Treatment C, presenting an approximate 4-fold increase in expression. The plant cuticle and cell wall inhibit the attempted penetration of microbial pathogens through a series of wall-associated defense reactions [45]. Pathogen hydrolases that target the plant cell wall are well-studied components of virulence, and it has been hypothesized that wall disassembly by the plant itself also contributes to susceptibility [46]. Thus, we posited that BDLA_00010249, the endo-β-1,3-glucanase, might be induced and involved in the cell-wall degradation of Chinese hickory, contributing to the activation of *B. dothidea*’s pathogenic state.

## 4. Conclusions

Chinese hickory trunk canker, caused by *Botryosphaeria dothidea*, poses a significant threat to the hickory industry. A critical aspect of its pathogenesis is the transition of *B. dothidea* from a latent to an overt infectious state, typically observed annually in spring when temperatures rise from 15 °C to 25 °C. However, the molecular mechanisms driving this crucial transition remain poorly understood. In this study, we simulated disease progression under controlled environmental conditions. Through co-expression network analysis and corresponding functional validation, we report that the pH-responsive transcription factor PacC is involved in the temperature-mediated latent infection transition of *B. dothidea*. This finding is particularly concerning given recent global warming trends: according to Hansen et al., global temperatures have surged by over 0.4 °C in the past two years [47], leading to an average temperature increase of 1.6 °C compared to the early part of the last century. Such warming trends are alarming because *B. dothidea* pathogenicity is readily activated when spring temperatures reach 20–25 °C, initiating host infection. Therefore, identifying key factors involved in *B. dothidea*’s latent-to-pathogenic transition is paramount for developing effective disease control strategies and enhancing the hickory industry’s resilience to global warming.

## 5. Methods and Materials

### 5.1. The Plant Material and Pathogens Used for the Inoculation Assays

The plant materials used in this study consisted of two-year-old seedlings of the Chinese hickory tree (*Carya cathayensis* Sarg.) from the ‘Zhelinshan 3’ cultivar. These seedlings were cultivated in growth chambers (Model RLD-450D-4, Yongzhou Ledian, Ningbo Ledian Instrument Manufacturing Co., Ltd., Ningbo, China) under conditions of 25 °C, 75% relative humidity, and a 12 h light/12 h dark photoperiod for 2 weeks. The hickory seedlings were grown in a sand–peat mixture. The *Botryosphaeria dothidea* strain BDLA16-7, isolated in our previous research [7,27], was cultured on potato dextrose agar (PDA) supplemented with 100 µg/mL kanamycin sulfate and maintained at 25 °C. The 5-day-old mycelium was harvested, and 1 cm mycelial agar discs were excised and used for wound inoculations on the trunks of the hickory seedlings. The inoculated seedlings were incubated at 25 °C for the first 3 dpi to ensure hyphal colonization within the host plant. The inoculated seedlings were divided after 4 dpi into the following three groups: Treatment A, Treatment B, and Treatment C. These groups were transferred to growth chambers maintained at 25 °C, 35 °C, and 15 °C, respectively, for five days. All treated seedlings were relocated at 9 dpi to growth chambers maintained at 25 °C for 7 days. Taken together, nine seedlings were used per replicate, with three biological replicates (a total of 27 seedlings).

### 5.2. RNA Extraction, Transcriptome Sequencing, and Differential Expression Analysis

The trunks of inoculated seedlings were collected for total RNA isolation using a TRIzol reagent (TaKaRa Inc., Dalian, China) following the manufacturer’s protocols. The sequencing library was generated using an Illumina True-Seq RNA Library Prep Kit (San Diego, CA, USA) according to the manufacturer’s protocol. The 150 bp paired-end reads of high-throughput data were generated using an Illumina HiSeq-2000 platform. The RNA sequencing data were deposited at the NCBI SRA website with the accession number PRJNA1137604.

The quality of the sequencing data was evaluated using the Trimmomatic software [48]. Clean data were acquired by eliminating adapters, poly-N sequences, and low-quality reads from the raw data. The data were then aligned to the annotated genome of the *Botryosphaeria dothidea* strain BDLA16-7 using HISAT2, as described in our previous publication [27]. The level of transcript or gene expression was quantified using the FPKM (fragments per kilobase of transcript per million mapped fragments) value, which was calculated using the StringTie software [49]. Differentially expressed genes (DEGs) were identified using the DESeq2 R package (version 1.48.1) [44] with a |log2Fold Change| ≥ 1 and a false discovery rate (FDR) of < 0.05.

### 5.3. The Construction of the Weighted Gene Co-Expression Network

Differentially expressed genes (DEGs) that were expressed in at least one treatment comparison were selected for the construction of a co-expression network using the WGCNA package within the R software (version 4.3.3). This employed a scale-free network construction method that identified gene clusters with highly correlated expression profiles. The WGCNA package was used to calculate the soft-threshold parameter β value (which was selected when the fitting curve first approached 0.9) to meet the prerequisite of scale-free network distribution. The β value was set to 9 in this study. Based on the β value, we inferred the Pearson correlation coefficients among the genes based on their FPKM values, transforming the correlation matrix into an adjacency one. This adjacency matrix was subsequently transformed into a topological overlap matrix (TOM), and the gene interaction network was constructed. The subsequent steps involved employing the dynamic tree cut algorithm to classify genes into modules according to their module eigengenes (MEs). Closely related modules were merged. We defined the threshold and parameters so that the module similarity threshold = 0.25 and the minimum number of genes in the module = 30; these were set to divide the modules.

### 5.4. Selection and Functional Enrichment Analysis of Target Gene Modules

The correlation coefficients between the module eigengenes and phenotypic data (temperature settings in Treatment A, Treatment B, and Treatment C) were calculated to estimate the module–trait associations and to investigate the gene modules linked to the latent infection phase of *Botryosphaeria dothidea* in Chinese hickory. The module with the highest correlation coefficient was selected as the target module.

### 5.5. The Identification and Annotation of Hub Genes in the Target Module

We calculated the gene significance (GS) value and module membership (MM) value for each DEG in the target module using the WGCNA R package to identify the key genes within the target module. DEGs with a GS value of > 0.2 and an MM value of > 0.8 were defined as hub genes. The functional annotation (including GO and Nr annotations) of the hub genes was performed using eggNOG v2.1.12 (http://eggnog-mapper.embl.de/, accessed on 5 January 2025). A GO enrichment analysis was performed using clusterProfiler R packages [50]; GO or KEGG pathway terms with an adjusted *p*-value ≤ 0.05 indicated significant enrichment. The visualization of enrichment terms was conducted using the ggplot2 R package (version 3.5.2). We calculated the degree of hub genes (which represented the number of edges associated with each hub gene) to confirm their high connectivity within the co-expression network. The calculation and visualization of degrees were conducted using the Cytoscape software, v 3.7.2 [51].

### 5.6. The Validation of the Hub Genes Using Quantitative Real-Time PCR

A total of 15 hub genes were selected for the RT-qPCR assay to validate their expression level. Primer pairs of the candidate hub genes were designed using Primer3 (https://primer3.ut.ee/, accessed on Nov 2024). The β-tubulin gene *B. dothidea* (BDLA_00007187) was used as the internal reference gene [24]. Samples subjected to Treatment C (25 °C for 3 days > 15 °C for 5 days > 25 °C for 7 days) were chosen for this study. RNA isolation was performed using a Qiagen RNeasy Mini Kit (Qiagen Inc., Valencia, CA, USA), followed by cDNA synthesis using a Superscript IV Reverse Transcriptase cDNA Synthesis Kit (TB Green^®^ Premix Ex Taq™ II, Takara Bio Inc., Kusatsu, Japan) with 2 µg of template RNA. All cDNA samples were then diluted to a concentration of 20 ng/µL in preparation for the qRT-PCR. Gene expression levels were determined through qRT-PCR using a Bio-Rad Real-Time PCR System and the SYBR green dye method. The expression levels of the selected hub genes were quantified using the 2^−∆∆CT^ method. Histograms were generated using GraphPad Prism 8. The significance (*p* < 0.05) and standard error (SE) were ascertained via Duncan’s new multiple range test.

### 5.7. Construction of Deletions and Complemented Mutants

The PacC transcriptional factor BDLA_00001555 was selected for the functional validation. Gene-deletion vectors were engineered using a double-joint PCR approach [50]. These knockout constructs were introduced into the protoplasts of the wild-type BDLA16-7 strain using a polyethylene glycol (PEG)-mediated protoplast transformation. The screening of potential gene-deletion mutants was performed using a PDA medium supplemented with 100 µg/mL hygromycin. Putative gene-deletion mutants were then verified using PCR.

Fragments of candidate hub genes containing the native promoter and an open reading frame (ORF) were amplified and fused by a double-joint PCR. The resulting PCR products were co-transformed with *XhoI*-digested pYF11 into *Saccharomyces cerevisiae* XK1-25, employing an Alkali-Cation Yeast Transformation Kit (MP Biomedicals, Solon, OH, USA). Recombinant plasmids were extracted from the transformed yeast cells using a Yeast Plasmid Extract Kit (Solarbio, Beijing, China) and subsequently propagated in the *Escherichia coli* strain DH5α. These vectors were then reintroduced into the gene-deletion mutants by PEG-mediated transformation.

## Figures and Tables

**Table 1:** Summary of alignment statistics in 9 libraries referring to *B. dothidea* genome.

Sample	Raw Reads	Clean Reads	Mapped Reads
A-3-rep1	33,063,322	28,434,457 (86%)	1,031,575 (3.12%)
A-3-rep2	33,100,103	29,459,092 (89%)	1,112,163 (3.36%)
A-3-rep3	33,093,090	28,129,127 (85%)	1,068,906 (3.23%)
A-8-rep1	37,309,993	32,459,694 (87%)	1,385,662 (3.71%)
A-8-rep2	36,218,469	32,958,807 (91%)	1,355,090 (3.74%)
A-8-rep3	35,317,069	29,666,338 (84%)	1,293,980 (3.66%)
A-15-rep1	38,881,872	34,216,047 (88%)	577,038 (1.48%)
A-15-rep2	37,450,144	33,705,130 (9%)	563,034 (1.50%)
A-15-rep3	36,578,848	30,360,444 (83%)	517,571 (1.41%)
B-3-rep1	33,663,537	28,614,006 (85%)	1,190,962 (3.54%)
B-3-rep2	33,746,234	29,359,224 (87%)	1,257,647 (3.73%)
B-3-rep3	33,754,092	30,041,142 (89%)	1,287,632 (3.81%)
B-8-rep1	39,204,555	32,931,826 (84%)	410,609 (1.05%)
B-8-rep2	41,068,181	35,318,636 (86%)	428,930 (1.04%)
B-8-rep3	36,002,844	33,122,616 (92%)	372,589 (1.03%)
B-15-rep1	35,088,723	30,878,076 (88%)	946,741 (2.70%)
B-15-rep2	36,922,243	30,645,462 (83%)	981,773 (2.66%)
B-15-rep3	40,480,611	34,408,519 (85%)	1,077,289 (2.66%)
C-3-rep1	33,210,243	28,892,911 (87%)	1,212,173 (3.65%)
C-3-rep2	33,421,176	29,744,847 (89%)	1,293,399 (3.87%)
C-3-rep3	33,514,076	28,822,105 (86%)	1,203,155 (3.59%)
C-8-rep1	39,289,045	33,002,798 (84%)	2,488,240 (6.33%)
C-8-rep2	34,610,850	31,495,874 (91%)	2,053,054 (5.93%)
C-8-rep3	36,050,384	31,724,338 (88%)	2,211,313 (6.13%)
C-15-rep1	38,819,866	34,937,879 (90%)	1,976,784 (5.09%)
C-15-rep2	37,800,346	31,374,287 (83%)	1,991,694 (5.27%)
C-15-rep3	37,947,859	32,255,680 (85%)	1,967,094 (5.18%)

## Data Availability

The original data presented in the study are openly available in the NCBI SRA website with accession of PRJNA1137604.

## Supplementary Materials

The following supporting information can be downloaded at https://www.mdpi.com/article/10.3390/jof11080580/s1, Figure S1. Principal component analysis (PCA) of RNA-seq data samples. Figure S2. Investigation of DEGs before co-expression network construction. (A) A dendrogram of all samples; (B) measurement of temperature conditions of each sample; threshold determination of co-expression network of soft threshold (β value) (C); and free topology scale (D). Figure S3: Quantitative real-time PCR verification of expression of PacC gene in wild-type strain (BDLA16-7), *PacC* mutant strain (Δ*PacC*) and complemented the strain (Δ*PacC*-C). Table S1. DEG identification for each comparison. Table S2. Expression level of DEG identified in each comparison (FPKM value). Table S3. Module assignment of DEGs. Table S4. Hub genes of module royalblue. Table S5. GO enrichment analysis for hub genes of the royal-blue module.
