# Supplementary material for: Co-Expression Network Analysis Suggests PacC Transcriptional Factor Involved in Botryosphaeria dothidea Pathogenicity in Chinese Hickory
Source: J Fungi (Basel). 2025 Aug 4;11(8):580. doi: 10.3390/jof11080580 (PMC12387645; doi:10.3390/jof11080580)
Supplement: Supplementary file 1 [file jof-11-00580-s001.zip › jof-3762515-Supplementary figures.pdf]

Supplementary Materials

Co-Expression Network Analysis Suggests PacC Transcriptional Factor Involved in *Botryosphaeria dothidea* Pathogenicity in Chinese Hickory

Dong Liang †, Yiru Jiang †, Wei Ai, Yu Zhang, Chengxing Mao, Tianlin Ma * and Chuanqing Zhang *

College of Advanced Agricultural Sciences, Zhejiang Agriculture and Forest University, Hangzhou 311300, China; liangdong0309@163.com (D.L.)

* Correspondence: czipotw@163.com (T.M.); cqzhang@zafu.edu.cn (C.Z.)

† These authors contributed equally to this work.

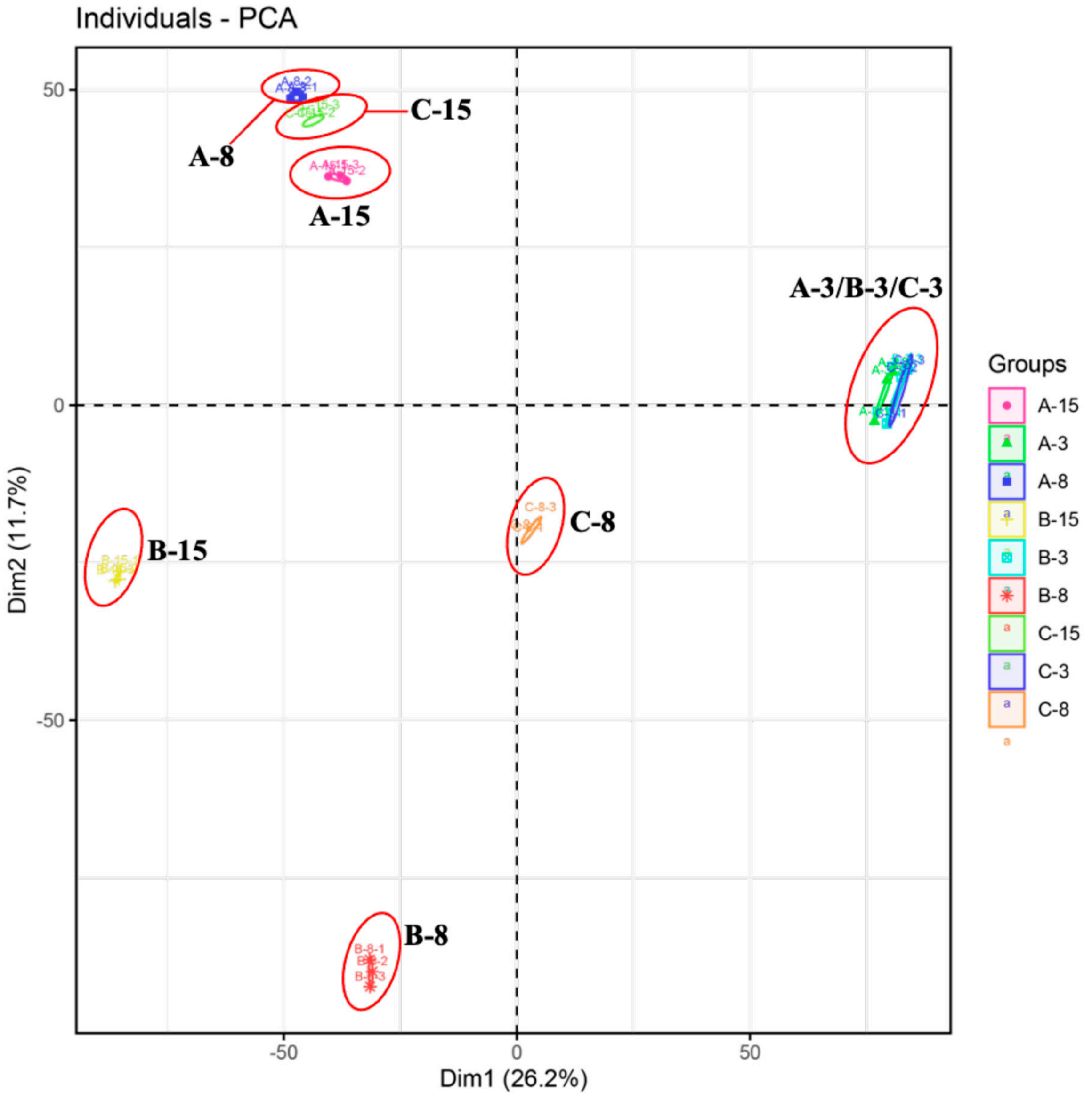

Figure S1. Principal component analysis (PCA) of RNA-seq data samples.

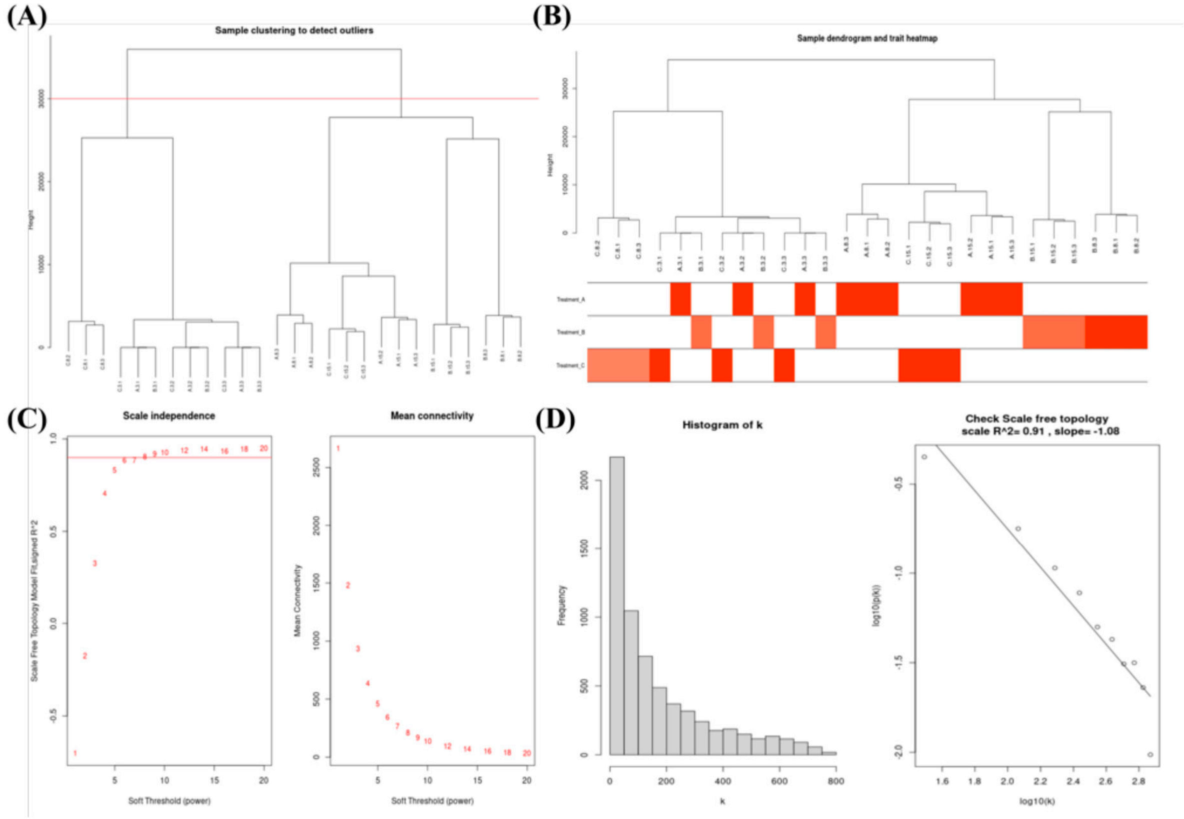

Figure S2. Investigation of DEGs before co-expression network construction. (A) A dendrogram of all samples; (B) measurement of temperature conditions of each sample; threshold determination of co-expression network of soft threshold (β value) (C); and free topology scale (D).

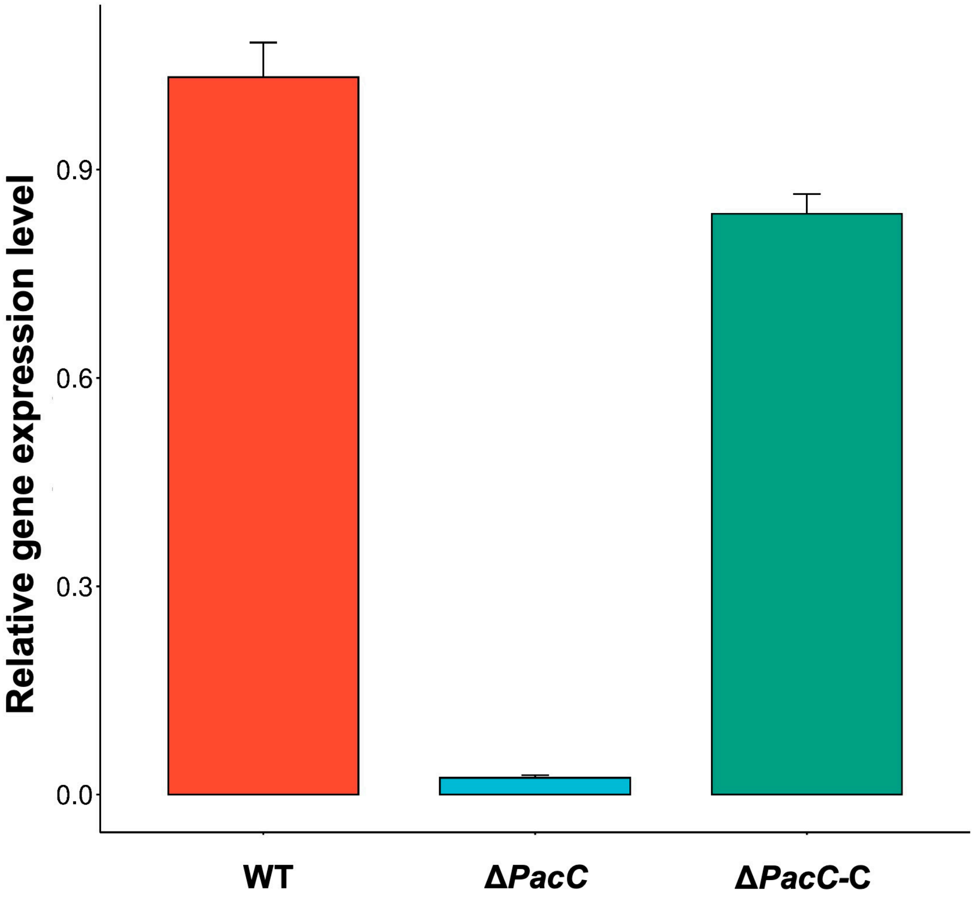

Figure S3. Quantitative real-time PCR verification of expression of PacC gene in wild-type strain (BDLA16-7), *PacC* mutant strain (Δ*PacC*) and complemented the strain (Δ*PacC*-C).
